# Polyphosphate-loaded silk fibroin membrane as hemostatic agent in oral surgery: a pilot study

**DOI:** 10.1186/s40729-023-00503-0

**Published:** 2023-11-17

**Authors:** Zohal Popal, Katrin F. Nickel, Michael Wöltje, Dilbar Aibibu, Christian Knipfer, Ralf Smeets, Thomas Renné

**Affiliations:** 1 Department of Anesthesiology, Center of Anesthesiology and Intensive Care Medicine, University Medical Center Hamburg-Eppendorf, Hamburg, Germany; 2 Institute of Clinical Chemistry and Laboratory Medicine, University Medical Center Hamburg-Eppendorf, Hamburg, Germany; 3 Institute of Textile Machinery and High-Performance Material Technology, TUD Dresden University of Technology, Dresden, Germany; 4 Department of Oral and Maxillofacial Surgery, University Medical Center Hamburg-Eppendorf, Hamburg, Germany; 5 Department of Oral and Maxillofacial Surgery, Division of Regenerative Orofacial Medicine, University Medical Center Hamburg-Eppendorf, Hamburg, Germany; 6 Center for Thrombosis and Hemostasis (CTH), Johannes Gutenberg University Medical Center, Mainz, Germany; 7 Irish Centre for Vascular Biology, School of Pharmacy and Biomolecular Sciences, Royal College of Surgeons in Ireland, Dublin, Ireland

**Keywords:** Anticoagulants, Fibroin, Hemorrhage, Hemostatic, Oral surgery, Polyphosphate, Silk, Thrombin

## Abstract

**Purpose:**

Post-interventional hemorrhage can result in serious complications, especially in patients with hemostatic disorders. Identification of safe and efficient local hemostatic agents is important, particularly in the context of an ageing society and the emergence of new oral anticoagulants. The aim of this in vitro study was to investigate the potential of silk fibroin membranes coated with the inorganic polymer polyphosphate (polyP) as a novel hemostatic device in oral surgery.

**Methods:**

Cocoons of the silkworm *Bombyx mori* were degummed and dissolved. Varying amounts of long-chain polyP (2–2000 µg/mm^2^) were adsorbed to the surface of silk fibroin membranes. Analysis of the procoagulant effect of polyP-coated silk membranes was performed using real-time thrombin generation assays in human plasma. Increasing concentrations of polyP (0.15–500 µg/ml) served as a positive control, while uncoated silk fibroin membranes were used as negative control.

**Results:**

PolyP-coated silk fibroin membranes triggered coagulation when compared to plasma samples and pure silk fibroin membranes. A polyP-dose-dependent effect of thrombin generation could be found with a maximum (ETP = 1525.7 nM⋅min, peak thrombin = 310.1 nM, time to peak = 9.8 min, lag time = 7.6 min.) at 200 µg/mm^2^ of polymer loading on the silk fibroin membrane surface.

**Conclusions:**

In this study, it was demonstrated that silk fibroin membranes coated with polyP have the potential to act as a promising novel hemostatic device.

**Graphical Abstract:**

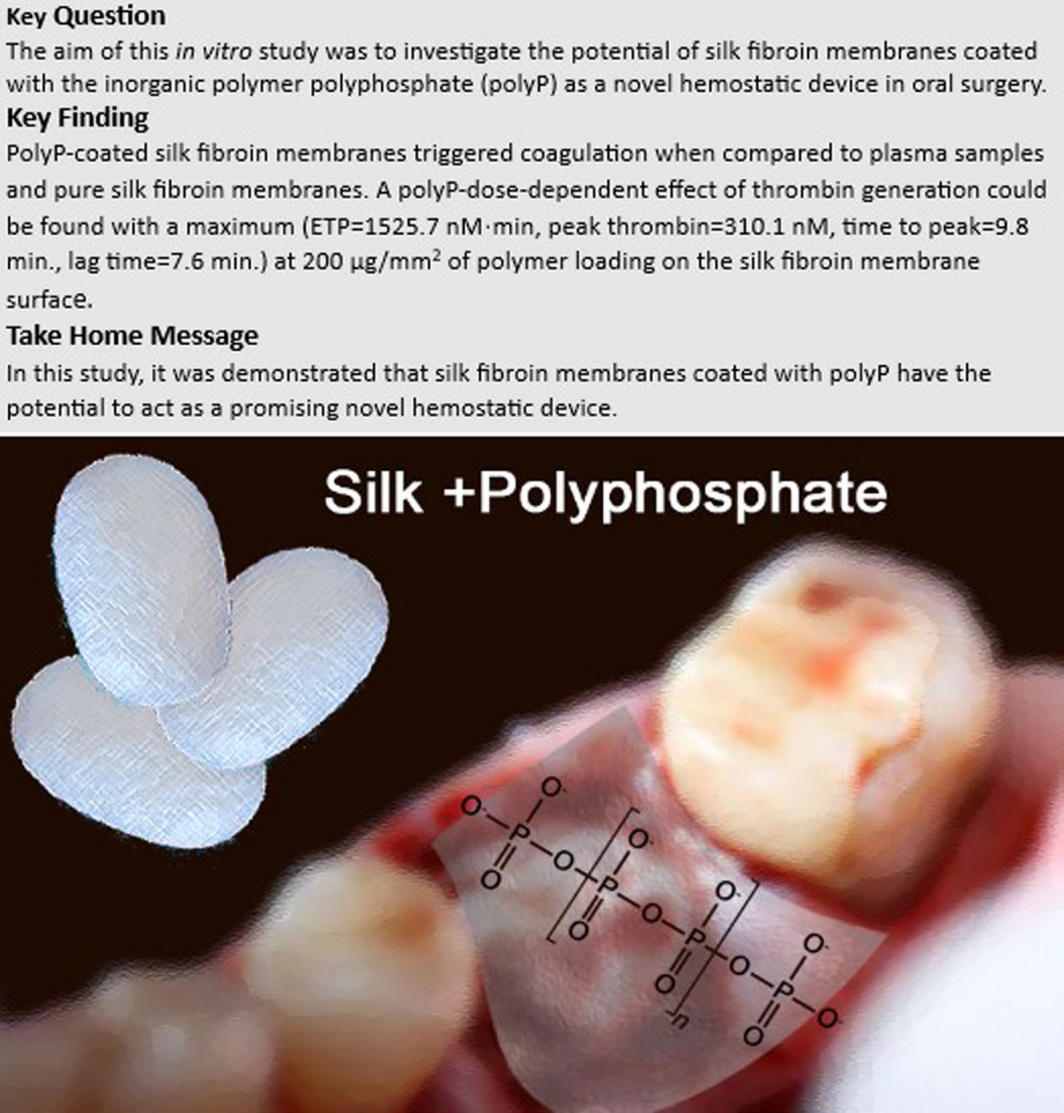

## Introduction

Hemostasis is a complex physiological response to prevent blood loss from injured vessels. Initially, platelets adhere to the vascular injury, become activated, and form a platelet plug that seals the injury (primary hemostasis). Fibrin produced by the blood coagulation systems stabilizes the clot (secondary hemostasis). The hemostatic machinery, which includes both pathways that interact simultaneously in the process of primary and secondary hemostasis, stops bleeding from minor wounds and contributes to wound healing [1]. However, severe trauma associated with major vascular injury can result in life-threatening blood loss [2]. In particular, patients with hereditary or acquired hemostatic disorders are at increased risk of bleeding and require immediate and specific treatment. Similarly, patients on anticoagulant medications have impaired hemostatic capacity and are prone to persistent local bleeding [3]. In recent years, new types of anticoagulants have been introduced. However, their impact on spontaneous and post-operative bleeding has not been fully elucidated. Therefore, in an ageing society where both the number of surgical interventions and the number of patients being treated with oral anticoagulants are increasing, efficient strategies for local support of hemostasis are needed. The rapid and efficient administration of potent topical medications for hemostasis is hence becoming increasingly important for surgical patients, especially during anticoagulant therapy [3]. Post-interventional bleeding is frequently observed as an adverse complication in oral surgery [4]. Oral wounds are particularly susceptible to bleeding due to dense blood supply, mechanical stress from chewing, breathing and articulation. In addition, they are exposed to fluids such as saliva and the physiological oral microbiome. Furthermore, the tissue of the upper gastrointestinal tract has a high fibrinolytic activity and thus an increased capability to dissolve already formed blood clots during hemostasis [5]. Overall, the specific oral environment has a strong influence on hemostasis and wound healing. The recently published guideline of the German Society for Dental, Oral and Maxillofacial Medicine (DGZMK) emphasizes the advantages of local topical hemostasis in oral surgery, especially for patients taking anticoagulant drugs [6]. In contrast, intervention with systemic hemostasis by administration of pro-coagulant or anti-fibrinolytic drugs remains under debate [6]. Treatment strategies for oral bleedings include bypass agents, such as recombinant factor VIIa (rFVIIa), activated prothrombin complex concentrates (aPCC, e.g., Factor VIII Inhibitor Bypassing Activity [FEIBA]), or fibrin sealants (fibrin glue). The latter reproduce the final stage of the coagulation cascade and subsequently the formation of a fibrin clot [7]. While these agents have potent hemostatic capacity and interfere with bleeding, the administration of current topical hemostatic medications is associated with an array of adverse side effects, including compromised local wound healing [8, 9], local inflammation [8–11], anaphylactic reactions [8, 11, 12], formation of heat that can lead to burn injuries [13], risk of disease transmission [14] and high costs [8, 9]. Overall, post-operative local hemostasis remains a challenge in patients undergoing oral surgery, especially while receiving anticoagulation therapy or when hemostatic abnormalities are present. To date, none of the currently available topical hemostatic agents meet the criteria for a low-risk, effective, and safe local hemostasis. Polyphosphate (polyP) is an inorganic, negatively charged polymer composed of orthophosphate residues linked by phosphoanhydride bonds. PolyP is abundant in nature, regulated by phosphate-homeostasis [15], non-immunogenic, degraded in plasma by endogenous phosphatases with a half-life of about 90 min, is inexpensive, and is easy to store and apply. Recently, the polymer has gained interest with regard to local hemostasis. PolyP is a procoagulant by several fibrin-forming mechanisms. In vivo, polyP contact-activates factor XII (FXII) to activated FXII (FXIIa) which in turn triggers the “intrinsic” pathway of blood coagulation [16–18]. Animal studies and experiments with human plasma have shown that the pharmacological inhibition of FXIIa and its activator polyP is associated with thromboprotection without increased clinical bleeding [19–22]. Moreover, in vitro studies suggest that polyP contributes to fibrin formation by accelerating thrombin-dependent activation of factor XI and factor V, as well as von Willebrand factor binding. It also decreases fibrinolysis and stabilizes the fibrin clot [23]. PolyP is produced in bulk form as a powder. For utilization as a local hemostatic agent in routine clinical practice, an improved method for defined application is required, e.g., a carrier material must be coated with polyP. In recent years, silk fibroin has gained importance in regenerative medicine research and is currently being investigated in various forms such as sponges, hydrogels, electrospun fibers, tubes or membranes [24]. Due to its mechanical versatility and high cytocompatibility [25–27] silk appears to be a suitable carrier for hemostatic agents. The aim of this feasibility study is to evaluate the potential of silk membranes coated with polyP for local hemostasis in vitro.

## Materials and methods

### Preparation of aqueous silk solution

Silk fibroin was isolated from cocoons of the larvae of the silk moth *Bombyx mori*. Silk cocoons were cut into small pieces and boiled for 30 min in 0.02 M Na_2_CO_3_. This process removes sericin from the silk fibroin fibers [24]. Then, silk fibroin fibers were dissolved in Ajisawa’s reagent at 60 °C for 4 h [28]. After complete dissolution of the silk fibroin fibers, the silk solution was dialyzed using a cellulose semi-permeable membrane (molecular weight cutoff: 10 kDa). The silk fibroin solution was dialyzed using a stepwise protocol published by Zheng et al. (2016) in which the dialysis tube was placed in 100 parts of 4 M urea solution for 3 h with slow stirring, followed by changing the solution to 100 parts of 2, 1 M urea and finally water for 3 h, respectively [29].

### Fabrication of the polyP-coated silk membranes

Varying amounts of long-chain polyP with an average chain length of > 1000 Pi (Phoskadent M, BK Giulini GmbH, Ladenburg, Germany) were homogeneously coated on the surface of 6-well culture plates (surface area: 9.6 cm^2^). Then, 1.8 ml of aqueous silk fibroin solution (9% weight by volume) was added to each well and during incubation for 48 h at room temperature in a safety cabinet, the water evaporated, leaving a membrane that physically tethered the polyP to the membrane surface.

### Blood collection

Peripheral venous blood was obtained from members of the study group and stored in 3.2% trisodium citrate (9:1 blood-to-citrate ratio). The tubes were centrifuged at 3000×*g* for 10 min twice at room temperature before platelet-poor plasma (PPP) was drawn off the upper portion using a pipette. The PPP was stored frozen at − 20 °C and thawed at 37 °C before use.

### Real-time thrombin formation

Thrombin formation in real-time was analyzed using the Calibrated Automated Thrombin Generation Assay (CAT) in a Fluoroscan Ascent fluorometer (Thermo Scientific, Waltham, MA, USA) equipped with a dispenser (Thrombinoscope BV, Maastricht, The Netherlands) as described [30]. The CAT method, developed by Hemker et al., uses a fluorogenic thrombin substrate, which allows real-time thrombin detection in platelet-poor and platelet-rich plasma samples [31]. The experiments were measured in a 96-well plate fluorometer. The Thrombin Calibrator (TC), dispensed into the first two wells together with plasma, was used as a reference for the Thrombinoscope to calculate the molar concentration of thrombin in our test wells against the calibrator. Thrombin formation was quantified using the Thrombinoscope software package (Version 3.0.0.29). After the measurement, the program expresses the results of thrombin activity. The area under the curve, i.e., the endogenous thrombin potential (ETP) indicates the total thrombin-forming capacity in nanomolar (nM) versus time, while the peak thrombin value indicates the maximum thrombin concentration reached. Time to peak (ttPeak) is defined as the minute the thrombin generation reaches peak height. Furthermore, the lag time defines the time until 1/6 of the peak height is reached and thus represents the initiation phase of coagulation. All data were collected from two independent experiments in duplicates and expressed as a mean value ± standard deviation (SD), unless otherwise indicated.

### PolyP membrane-driven thrombin generation

Silk fibroin membranes coated with polyP were cut into 3 × 3 mm pieces with a scalpel. 80 µl PPP, 10 µl MP Reagent (Thrombinoscope BV, 4 µM final phospholipid concentration) and 10 µl PBS with pieces of silk membranes coated with increasing polyP concentrations (2 µg/mm^2^, 6 µg/mm^2^, 20 µg/mm^2^, 60 µg/mm^2^, 200 µg/mm^2^, 600 µg/mm^2^ and 2000 µg/mm^2^) were added into each well. After addition of 20 µl FluCa (containing 2.5 nM fluorogenic substrate and 100 mM calcium chloride), a solution automatically dispensed by the machine, which initiates the coagulation reaction, the final volume amounted to 120 µl in each well. Uncoated silk fibroin membranes functioned as negative controls. For positive control we used 80 µl PPP together with 10 µl phospholipids and stimulated it with 10 µl polyP (Phoskadent M) in solution, leading to final concentrations of 0.15 µg/ml, 0.5 µg/ml, 1.5 µg/ml, 5 µg/ml, 15 µg/ml, 50 µg/ml, 150 µg/ml to 500 µg/ml.

### Scanning electron microscopy (SEM)

PolyP distribution and silk fibroin membrane structure were evaluated using a Quanta™ 250 FEG ESEM scanning electron microscope (FEI Company, USA).

## Results

**Fig. 1:**
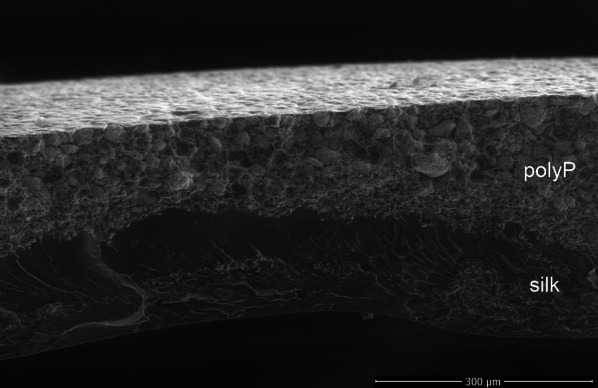
Cross-section of a polyP-functionalized silk fibroin membrane (polyP: 200 µg/mm^2^)

The distribution of polyP within the silk fibroin membrane was investigated by scanning electron microscopy (SEM). As shown in Fig. 1, there is a polyP phase with a smooth surface on the upper side of the functionalized membrane. The lower part of the membrane consists of pure silk fibroin protein. The polyP particles are physically bound to the membrane by the silk fibroin protein solution after the aqueous part is evaporated. In this way, a two-phase membrane is formed, consisting of a polyP layer on one side and a pure silk layer on the other side.

**Fig. 2:**
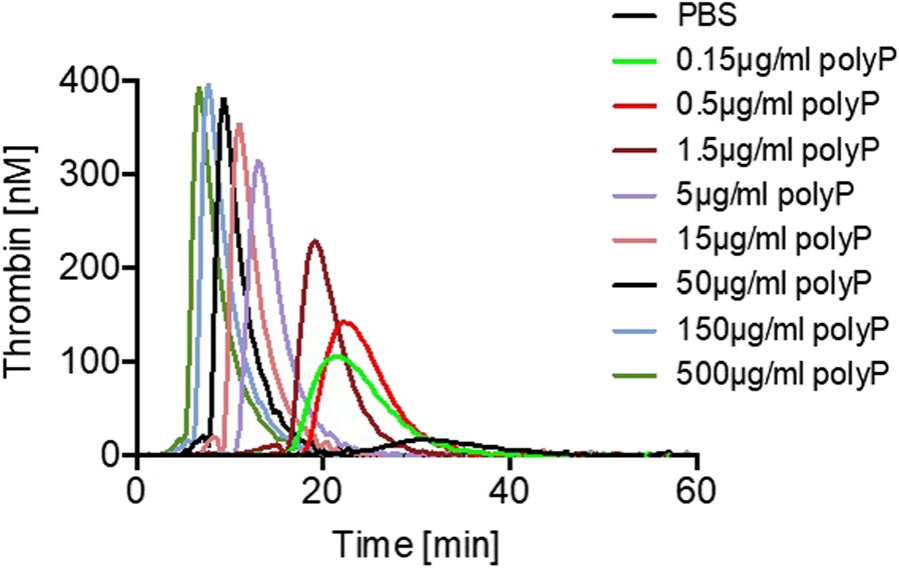
Real-time thrombin generation initiated by pure polyP increasing concentrations in human plasma. (*n* = 1 in duplicates)

**Fig. 3:**
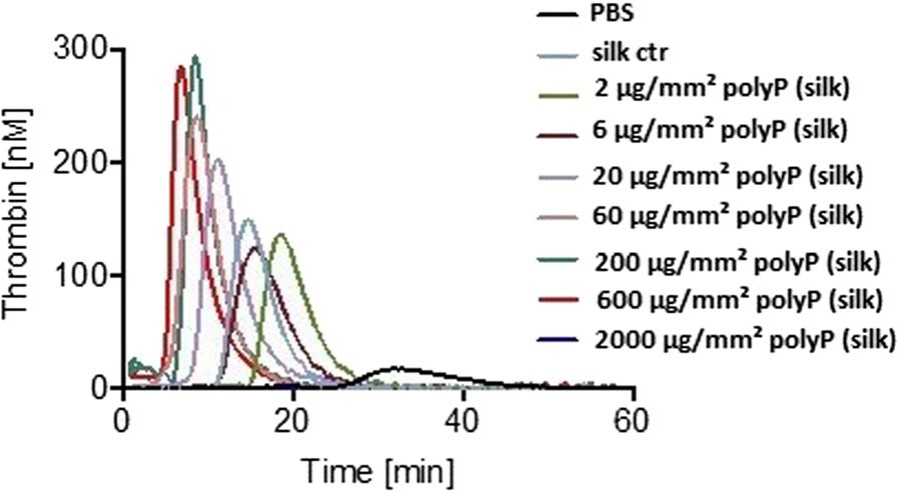
Thrombin generation in real time triggered by polyP-functionalized silk fibroin membranes (*n* = 2, in duplicates)

To analyze the procoagulant activity of polyP-coated silk fibroin membranes, we performed real-time thrombin generation assays in human plasma in the absence or presence of pure polyP and silk fibroin membranes coated with increasing concentrations of polyP. First, we stimulated plasma with pure polyP in increasing concentrations. Concentrations of 150 µg/ml polyP potently initiated thrombin generation leading to an ETP of 1544.3 nM⋅min, peak thrombin of 382.9 nM, ttpeak of 8 min and lag time of 6.3 min. In contrast, polyP with the lowest concentration tested (0.15 µg/ml) generated an ETP of 980.6 nM⋅min, peak thrombin of 107 nM, time to peak of 21.7 min and lag time of 17 min (Fig. 2, Table 1). PolyP-coated silk fibroin membranes with a polyP surface-amount of 6–600 µg/mm^2^ were procoagulant compared to pure silk fibroin membranes (Fig. 3). Higher quantities of polyP (2000 µg/mm^2^) did not lead to a stronger procoagulant effect in vitro. The procoagulant activity even decreased with higher polyP amounts on the membrane surface, most likely through complex formation with calcium ions, which effectively interrupts thrombin production. The thrombin generation triggered by polyP-coated silk fibroin membranes reached its maximum at a polyP amount of 200 µg/mm^2^ (ETP of 1525.7 nM⋅min), before it decreases with 600 µg/mm^2^ (ETP of 1339.3 nM⋅min). Figure 3 demonstrates thrombin generation in silk fibroin membrane-induced plasma under increasing polyP amounts on silk fibroin membrane surfaces.

**Table 1:** PolyP-driven thrombin generation of polyP control

	0.15	0.5	1.5	5	15	50	150	500
	µg/ml polyP							
ETP (nM⋅min)	980.6 ± 201	1108.9 ± 75	1246.8 ± 79	1398.5 ± 76	1398.9 ± 44	1453.9 ± 23	1544.3 ± 55	1522.4 ± 3.1
Peak thrombin (nM)	107 ± 31	143 ± 14	236.6 ± 48	316.4 ± 17	351.7 ± 11	367.6 ± 10	382.9 ± 18	374.6 ± 5.3
Time to peak (min)	21.7 ± 2.3	22.5 ± 0.5	19.5 ± 1.2	13.2 ± 1.5	11 ± 1	9.7 ± 0	8 ± 0.3	7.2 ± 0.2
Lag time (min)	17 ± 2	18.7 ± 0.7	16.7 ± 1.7	11 ± 1.3	9.2 ± 1.2	8 ± 0	6.3 ± 0.3	5.5 ± 0.2

Comparison of thrombin formation in plasma stimulated by increasing concentrations of pure polyp

Values are expressed as mean ± SD, *n* = 1 in duplicates

Thrombin generation was increased in plasma samples with polyP-coated silk fibroin membranes compared to buffer-treated normal plasma (Table 2). Functionalized silk fibroin membranes with a polyP amount of 200 µg/mm^2^ initiated the highest amount of thrombin reaching an ETP of 1525.7 nM⋅min and peak thrombin of 310.1 nM (Fig. 3). Consistently, lag time (7.6 min in 200 µg/mm^2^ polyP-coated membranes vs 23.3 min in buffer-treated PPP) was shortened by 67% while time to peak (9.8 min in 200 µg/mm^2^ polyP-coated membranes vs. 28.8 min in PPP) was shortened by 66%. Accordingly, peak thrombin (310.1 nM in 200 µg/mm^2^ polyP-coated membranes vs. 52.3 nM in PPP) was significantly increased by 5.96-fold and total thrombin, i.e., ETP (1525.7 nM⋅min in 200 µg/mm^2^ polyP-coated membranes vs. 519.2 nM⋅min in PPP) was also increased by 2.94-fold, in samples compared to plasma controls (Table 2).

**Table 2:** PolyP-driven thrombin generation of functionalized silk fibroin membranes

	Plasma with buffer (PPP)	Silk control	µg/mm^2^ silk + polyP						
			2	6	20	60	200	600	2000
ETP (nM⋅min)	519.2 ± 413	955.5 ± 119	970.6 ± 170	1098 ± 310	1279.9 ± 183	1391.2 ± 160	1525.7 ± 227	1339.3 ± 9	−1
Peak thrombin (nM)	52.3 ± 50	198.4 ± 70	175.9 ± 53	207.8 ± 119	246.2 ± 51	267.3 ± 38	310.1 ± 27	243.4 ± 56	−1
Time to peak (min)	28.8 ± 7	15.3 ± 1.4	17.1 ± 5	13.1 ± 6	11.8 ± 1.8	10.2 ± 0.5	9.8 ± 0.7	8.9 ± 0.6	−1
Lag time (min)	23.3 ± 6	12.8 ± 0.7	14 ± 4	10 ± 4	9.1 ± 1.5	7.4 ± 0.1	7.6 ± 0.6	6.8 ± 0.4	−1

Comparison of thrombin formation in normal platelet-poor plasma, silk fibroin membrane-induced plasma and polyP-coated silk fibroin membrane-induced plasma

PPP, platelet-poor plasma; silk + polyP, 3 × 3 mm silk fibroin membranes coated with polyp

Values are expressed as mean ± SD, *n* = 2 in duplicates

For negative control, plasma was stimulated with uncoated silk fibroin membranes. The results of pure silk fibroin membranes compared to 200 µg/mm^2^ silk + polyP were as follows: respectively, lag time (7.6 min in 200 µg/mm^2^ polyP-coated membranes vs 12.8 min in silk fibroin controls) was shortened by 41% and time to peak (9.8 min in 200 µg/mm^2^ polyP-coated membranes vs. 15.3 min in silk fibroin controls) was shortened by 36%. Accordingly, peak thrombin (310.1 nM in 200 µg/mm^2^ polyP-coated membranes vs. 198.4 nM in silk controls) was increased by 1.57-fold and total thrombin, i.e., ETP (1525.7 nM⋅min in 200 µg/mm^2^ polyP-coated membranes vs. 955.5 nM⋅min in silk fibroin controls) was also increased by 1.6-fold, in samples compared to uncoated silk fibroin membranes (Table 2). Both uncoated silk fibroin and buffer-treated normal plasma showed lower total (endogenous thrombin potential) and maximum (peak) thrombin formation, and extended time to peak and lag time, compared to polyP-coated silk fibroin membranes **(**Table 2, Fig. 3). In turn, uncoated silk fibroin alone shows a low procoagulant activity, compared to buffer-treated plasma samples (Table 2, Fig. 3).

## Discussion

The FXII activator polyP is biocompatible, inexpensive, non-immunogenic, and degradable by endogenous phosphatases. [32]. PolyP initiates fibrin formation in plasma treated with anticoagulants [33] and normalizes defective fibrin formation in plasma samples from patients with inherited platelet disorders [16], suggesting that the polymer could be used as an hemostatic agent. Indeed, the addition of synthetic polyP with a chain length of 75 phosphate subunits has been shown to reduce clotting times in plasma samples from hemophilia A and B patients [33] and in PRP from individuals with Hermansky–Pudlak syndrome, suggesting that the polymer may restore defective coagulation ability in these disorders. Several in vivo and in vitro experiments have demonstrated the critical role of the polyP/FXII- mediated coagulation in thromboembolic disorders [22, 34–36]. In addition to polyP, multiple other FXII contact activators have been identified such as RNA, collagen, and neutrophil cellular traps (NETs) [32]. Conversely, polyP contributes to the procoagulant activity of NETs [37]. However, the specific mechanisms of FXII contact activation are still unclear. Deletion mutants have recently shown that polyP activates FXII by binding to its proline-rich domain (PR-III) [32]. Although the PR-III region is essential for surface-induced activation of FXII, its absence still allows protease-mediated FXII activation in solution. Additionally, the transmembrane protein XPR1 (xenotropic and polytropic retrovirus receptor 1) has been shown to function as a major phosphate exporter in platelets and thus plays a significant role in the regulation mechanisms of phosphate hemostasis and thrombosis formation [15].

In vitro experiments have shown that the ability to activate FXII increases with the chain length of synthetic polyP [38], however, in vivo polyP forms Ca^2+^-rich nanoparticles that are insoluble [18], and upon release remain retained on the plasma membrane surface of platelets, platelet-derived microparticles and cancer-released extracellular vesicles. These natural Ca^2+^/polyP aggregates initiate the procoagulant and proinflammatory FXII-driven contact system independently of the chain length of the individual polyP polymer packed into nanoparticles. Supporting the hypothesis that polyP functions via activation of FXII, an array of polyP inhibitors interfere with thrombosis while sparing hemostasis thereby mimicking the effect of FXII neutralizing agents, recently reviewed in [39].

In order to apply and localize polyP to wounds without the risk of being washed out by wound secretions and blood, a support device is required to keep the polyP at the wound site. The results of the experiments presented support the hypothesis that a silk fibroin matrix is a promising candidate for such a support device.

Results from a mouse injury model showed that silk fibroin can activate the “intrinsic” coagulation pathway and thus reduce bleeding time [40]. Consistent with these results, silk fibroin as a control without polyP is already a mild procoagulant (Fig. 3). The prospects of silk fibroin scaffolds in combination with oxidized cellulose have been investigated with respect to their wound healing promoting properties [41]. Both properties provide an essential basis for the present approach. Evaluation of the procoagulant activity of silk fibroin membranes functionalized with polyP in vitro is a key factor in assessing further perspectives in this field. In the present pilot study, silk fibroin membranes were coated with increasing amounts of polyP and their procoagulant potential was investigated in vitro. The results of the study suggest that the procoagulant effect depends on the amount of polyP used as a coating for the silk fibroin carrier. PolyP-coated silk fibroin membranes show an increase in thrombin generation with increasing amounts of polyP. The dose-dependent thrombin generation with polyP-coated silk fibroin membranes reached its maximum at a polyP amount of 200 µg/mm^2^. However, higher polyP quantities do not necessarily imply a higher procoagulant activity. The results of pure polyP compared to the silk fibroin samples showed that polyP-coated silk fibroin membranes with an amount of 200 µg/mm^2^ polyP generate similar amounts of thrombin (ETP 1525.7 nM⋅min, peak 310.1 nM) and thus, have a procoagulant effect similar to 150 µg/ml pure polyP (ETP 1544.3 nM⋅min, peak 382.9 nM) (Tables 1 and 2). The preliminary results of the current study cannot quantify the exact amount of polyP presented on the surface of the silk fibroin carrier membrane, and thus no information can be obtained about the active polyP molecules in the coating that lead to thrombin generation. Practically, this study cannot quantify the effective amount of the active polyP molecules attached to the silk fibroin membrane surface. Nevertheless, the proposed method allows a homogenous distribution of polyP in silk fibroin up to 600 µg/mm^2^, while higher amounts lead to an inhomogeneous distribution. Recent studies have demonstrated the presence of polyP on the surface of activated platelets, stored together with Ca^2+^-ions, showing release mechanisms and deposition of polyP [18]. Thus, only small amounts of the poorly soluble calcium-polyP complexes are released from the platelet plasma membrane, while the majority remains attached [18, 42]. Further dose-finding studies are needed to quantify the optimal amount for homogeneous and efficient polyP coating of the silk fibroin carrier membrane.

In conclusion, we have demonstrated the prospect of polyP-functionalized silk fibroin membranes as a promising straightforward approach for local hemostasis in vitro. By coating silk fibroin membranes with polyP, the potential for an innovative local wound dressing for hemostasis in situ has been explored and offers good prospects for further investigation.

## Data Availability

Z.P and R.S. declare that all data supporting the findings of this study are available within the article and from R.S. upon request.
